# Autism, Intellectual Disability and Suicide Risk in Adolescent Psychiatric Emergencies: A Two-Year Retrospective Cohort Study

**DOI:** 10.3390/brainsci16030250

**Published:** 2026-02-24

**Authors:** Maria Giulia D’Acunto, Cristina Di Sarno, Francesca Lenzi, Francesca Liboni, Marika Ricci, Antonio Narzisi, Gabriele Masi, Maria Mucci

**Affiliations:** Department of Developmental Neuroscience, IRCCS Stella Maris Foundation, 56128 Calambrone, PI, Italy; cristina.disarno@ausl.bologna.it (C.D.S.); francesca.lenzi@fsm.unipi.it (F.L.); francesca.liboni@aslto3.piemonte.it (F.L.); marika.ricci@fsm.unipi.it (M.R.); gabriele.masi@fsm.unipi.it (G.M.); maria.mucci@fsm.unipi.it (M.M.)

**Keywords:** adolescents, neurodevelopmental disorders (NDDs), autism spectrum disorder (ASD), borderline intellectual functioning (BIF), intellectual disability (ID), psychiatric emergencies, suicidality

## Abstract

Background: Adolescents with neurodevelopmental disorders (NDDs), particularly autism spectrum disorder (ASD) and borderline intellectual functioning/intellectual disability (BIF/ID), represent a clinically complex population in psychiatric emergency settings, with unclear contributions to acute psychopathology and suicide risk. Aims: This study examined whether ASD and BIF/ID differentially influence clinical severity, psychopathological profiles, and suicidality in adolescents admitted for psychiatric emergencies, comparing high-functioning ASD, ASD with cognitive impairment, and adolescents without NDDs. Methods: We conducted a retrospective, single-center cohort study including 206 consecutive patients aged 11–17 years admitted to a psychiatric emergency unit between January 2022 and December 2023. Patients were stratified into four groups: ASD (ASD-HF; ASD-BIF/ID), BIF/ID and N-ASD/N-BIF/IDClinical severity, global functioning, psychiatric diagnoses, adverse childhood experiences, emotional dysregulation, and suicidality were assessed using standardized diagnostic and behavioral measures. Group comparisons were performed to identify predictors of suicidality. Results: ASD-BIF/ID patients exhibited the lowest global functioning, whereas ASD-HF adolescents showed functioning comparable to controls. Suicidal ideation and behaviors were significantly more frequent in ASD-HF. BIF/ID was associated with greater behavioral impairment and lower suicidality. Conclusions: ASD and BIF/ID may differentially shape psychiatric emergency presentations. Adolescents with high-functioning ASD showed a higher prevalence of suicidality in this specific clinical context. Limits: This study is limited by its cross-sectional, single-center, and retrospective design, small and uneven subgroup sizes, and assessment tools not specifically validated for autistic or intellectually disabled populations. The high prevalence of bipolar spectrum disorders may reflect referral bias. Despite these limitations, adolescents with high-functioning ASD exhibited elevated suicidality, underscoring the importance of risk assessment adapted to cognitive and diagnostic profiles.

## 1. Introduction

During the SARS-CoV-2 public health emergency and the subsequent post-pandemic period, a marked increase was reported in emergency department (ED) presentations among adolescents for various psychopathological conditions, including mood disorders, behavioral disorders, psychosis, and substance use disorders [1]. Among individuals aged 6 to 24 years, the proportion of ED visits related to mental health concerns increased from 7.7% to 13.1%, with an average annual percentage increase (AAPC) of 8.0%.

Numerous studies have shown that pediatric clinical populations with neurodevelopmental disorders experienced more severe affective and anxiety symptoms during the pandemic compared to neurotypical individuals [2,3].

Multiple studies have also identified the disruption of daily routines, limited access to schools, and reduced availability of specialized social services as key contributing factors to the observed changes in behavior, emotional regulation, and overall mental health in these patients [4,5,6,7,8,9,10].

Historically, research on suicidality has often excluded individuals with neurodevelopmental disorders [11], potentially limiting current knowledge regarding suicide risk in this population.

A recent review [12] aimed to identify studies investigating the association between neurodevelopmental disorders (NDDs) and suicide risk. Findings encompassed children and adolescents diagnosed with Attention-Deficit/Hyperactivity Disorder (ADHD), autism pectrum isorder (ASD), and Tourette Syndrome, highlighting both general risk factors—such as depression, adverse childhood experiences (ACEs), and emotional dysregulation—and disorder-specific risk factors, including camouflaging behaviors in individuals with ASD or impulsive behaviors in youth with ADHD. The review further proposed a theoretical model wherein emotional dysregulation and adverse childhood experiences (environmental factors) may contribute directly and indirectly to suicidality, largely through the development of depressive symptoms. Although depressive symptomatology consistently emerges as a key correlate of suicidal behavior across both neurodevelopmental and general pediatric populations [13], emotional instability and subthreshold or underrecognized bipolar features may warrant further consideration.

Adolescents with autism spectrum disorder without cognitive impairment may represent a clinically complex subgroup.

Previous studies have reported higher rates of hospital admissions among autistic youth along with greater functional impairment than non-autistic adolescents with psychiatric diagnoses [14,15,16].

A 2021 study reported that ASD patients presenting to psychiatric emergency services exhibit a higher prevalence of neuropsychiatric comorbidities, especially Attention-Deficit/Hyperactivity Disorder (ADHD), intellectual disability, epilepsy, and other psychiatric disorders encompassing both externalizing and internalizing symptoms [17].

Growing evidence also indicates elevated rates of suicidal ideation, suicide attempts, and suicide deaths among individuals with ASD [18]. A systematic review reported that approximately 25% experienced suicidal ideation and 8% had attempted suicide [19]. Some studies have documented differences in the characteristics and lethality of suicide methods used by individuals with ASD, which may partially account for differences in suicide outcomes reported across studies [20,21].

Suicide risk appears to be higher among individuals with high-functioning ASD and has been associated with higher cognitive functioning and educational attainment. Greater cognitive abilities may be linked to increased awareness of social difficulties, which has been associated with increased psychological distress in some individuals [18].

A pilot study published in 2020 [22], using a clinical database of 52 adolescents with bipolar disorder [BD], compared patients with comorbid ASD and suicidality (BD-ASD-S) to those with ASD without suicidality (BD-ASD-noS), finding that higher cognitive functioning was linked to an increased suicide risk among individuals with ASD.

In contrast, individuals with intellectual disability and borderline intellectual functioning have received comparatively less attention in suicidality research. Early hypotheses suggested that lower observed suicidality in adolescents with cognitive impairment may reflect methodological and clinical factors influencing its detection [23]. However, subsequent studies have shown that individuals with intellectual disability are frequently exposed to factors associated with increased suicide risk [24].

Compared to those without disabilities, young people with ID are more frequently subjected to abuse, neglect, social disadvantage, challenging family circumstances, stigma, and peer exclusion. They also experience higher rates of depressive symptoms [25]. Additionally, psychopathological symptoms in individuals with intellectual disability are often exacerbated by their vulnerability and deficits in cognitive and emotional coping mechanisms, which hinder their ability to adapt to their environment [12,25].

Given the increasing prevalence of neurodevelopmental disorders worldwide, further research in this area is warranted.

More specifically, despite the recognized role of psychiatric comorbidity as a major risk factor for suicidal ideation and attempts, the psychopathological profiles of these patients remain insufficiently understood. Although psychiatric assessment in specialized hospital settings is essential for identifying suicide risk and associated risk and protective factors in adolescents with neurodevelopmental disorders, further research is needed to clarify how the specific characteristics of each neurodevelopmental disorder influence psychiatric crises and suicidality.

ASD and BIF/ID are both neurodevelopmental conditions frequently associated with complex psychiatric profiles, including difficulties in emotional regulation, communication, and coping with stressors. These challenges may increase vulnerability to acute psychiatric crises and suicidal behaviors, especially during adolescence. However, ASD and BIF/ID can manifest in different ways and may contribute to psychiatric emergencies through distinct mechanisms.

The primary aim of the present study was to compare the prevalence and clinical correlates of suicidality across diagnostic subgroups.

The secondary aims were to [a] distinguish the specific clinical impact of ASD from that of BIF/ID; [b] explore whether high-functioning individuals with ASD differ from those with ASD and cognitive impairment; and [c] compare both groups to patients without these neurodevelopmental conditions. These secondary analyses should be considered exploratory and hypothesis-generating.

## 2. Methods

### 2.1. Sample

Inclusion criteria: In this observational, retrospective, cohort study, we included a consecutive, unselected sample of adolescents aged 11–18 years admitted to the Psychiatry Emergency Department between 1 January 2022, and 31 December 2023.

Exclusion criteria: Participants were excluded if they were younger than 11 years or older than 18 years at the time of admission; were not admitted to the Psychiatry Emergency Department during the study period (1 January 2022–31 December 2023); had incomplete, missing, or irretrievable clinical records that prevented extraction of the variables of interest; or explicitly objected to the secondary use of their personal data for research purposes, where such objection was recorded in accordance with applicable data protection regulations.

All admissions during the study period were screened for eligibility. Only the first admission for each patient was included in the analysis.

Informed consent was not required for this retrospective study under Italian law. Personal data may be used for research purposes pursuant to Article 110-bis(4) of the Italian Personal Data Protection Code, which allows public and private IRCCS (Scientific Institutes for Research, Hospitalization and Healthcare) to repurpose clinical data for research, provided that the safeguards outlined in Article 89 of the GDPR are respected.

The study was approved by the Ethics Committee of Hospital Meyer, Florence, Italy (code AP-NDD-RS) on 27 March 2025, and conducted in accordance with the Declaration of Helsinki.

The sample characteristics are reported in Table 1.

The entire cohort (N = 206) was divided into four groups:

- ASD (N = 41): Patients with autism spectrum disorder.
  ○ ASD-HF (N = 22): Patients with high-functioning autism spectrum disorder.
  ○ ASD BIF/ID (N = 19): Patients with autism spectrum disorder + Borderline intellectual functioning or Intellectual disability.
- BIF/ID (N = 32): Patients with Borderline intellectual functioning or Intellectual disability.
- N-ASD-N-BIF/ID (N = 133): Patients without Autism spectrum disorder or Borderline intellectual functioning/Intellectual disability.

### 2.2. Measures

Data were collected on the presence of established suicide risk factors, including a family history of psychiatric disorders, depression, suicide attempts, completed suicides, and adverse life events (e.g., trauma or abuse, high-conflict parental separation or divorce, bereavement, bullying, and self-injurious behaviors).

Additional information was obtained from the assessment carried out as part of routine clinical practice, using the following validated and standardized instruments:

- The Clinical Global Impression—Severity (CGI-S; [26]) scale is a clinician-rated measure that assesses the overall severity of a patient’s condition. Scores range from 0 to 7, with higher scores indicating greater severity.
- The Children’s Global Assessment Scale (C-GAS; [27]) is a clinician-rated global measure of overall functioning, ranging from 0 to 100, with higher scores indicating better functioning.
- The Kiddie Schedule for Affective Disorders and Schizophrenia for School-Age Children) (K-SADS-PL) [28] is a semi-structured diagnostic interview for psychopathological disorders (past and present) within an age range of 6 to 17 years according to DSM-IV criteria, administered to patients and parents by child psychiatrists specifically trained in the use of this interview.
- The Columbia Suicide Severity Rating Scale (C-SSRS; [29]) assesses the severity of suicidal ideation and behavior. A score of 3 or higher indicates clinically significant suicidal risk. The C-SSRS is recommended by the Centers for Disease Control and Prevention (CDC) and the U.S. Food and Drug Administration (FDA) for evaluating adolescents at high risk of suicide.

### 2.3. Statistical Analyses

All statistical comparisons were performed using SPSS version 19.0 for Windows.

Statistical analyses included both parametric tests (univariate ANOVA) and non-parametric tests (Chi-square test), depending on the nature of the variables considered [quantitative or qualitative]. The Bonferroni correction was applied for multiple simultaneous comparisons for quantitative variables. Z test was performed for multiple simultaneous comparisons for qualitative variables. A significance level of 0.05 was adopted. Results with a Pearson *p*-value less than 0.01 were considered highly significant.

## 3. Results

Group comparisons were performed on qualitative (gender) and quantitative clinical features (age, mean hospitalization duration, CGI, C-GAS), as summarized below.

Regarding gender, a significant difference was observed between the ASD and BIF/ID groups and the N-ASD/N-BIF/ID group, with a predominance of females in the N-ASD/N-BIF/ID group (Χ^2^(2, N = 206) = 15.82, *p* < 0.001). When the ASD group was further subdivided into ASD-HF and ASD-BIF/ID, the difference remained significant between the ASD-BIF/ID and N-ASD/Non-BIF/ID groups (Χ^2^(3, N = 206) = 22.3, *p* < 0.001).

For global functioning, significant differences were observed in C-GAS scores. The ASD group had a mean score of 33.75 (±10.45), which was significantly lower than the N-ASD/N-BIF/ID group (37.98 ± 8.17) (F(2, N = 206) = 3.27, *p* = 0.04). When subdividing the ASD group into ASD-HF and ASD-BIF/ID groups and comparing across all four groups, the ASD-BIF/ID subgroup showed significantly lower functioning (31.16 ± 10.99) than the N-ASD/N-BIF/ID group (37.98 ± 8.17) (F(3, N = 206) = 3.39, *p* = 0.019). ASD-HF patients did not differ significantly from N-ASD/Non-BIF/ID patients.

Further analyses were performed to compare the prevalence of suicidal ideation, suicide attempts, and the occurrence of non-suicidal self-injury [NSSI] across groups, as documented in clinical history, clinical interviews, or assessed via rating scales (Table 2 and Table 3).

No statistically significant differences were found between groups regarding the presence of NSSI.

A significant difference in suicidal ideation was observed between BIF/ID (21.9%) and N-ASD-N-BIF/ID (48.9%) (Χ^2^ (2, N = 206) = 9.68, *p* = 0.008). A significant difference was observed in suicidal behaviors between both ASD (19.5%) and BIF/ID [21.9%] groups and N-ASD-NBIF/ID (48.9%) (Χ^2^ (2, N = 206) = 11.28, *p* = 0.004).

When subdividing ASD population, ASD-HF demonstrated a significantly greater proportion of patients with suicidal ideation (45.5%) and suicide attempts (31.8%) compared to the ASD--BIF/ID group (15.8% and 5.3%, respectively) [suicidal ideation (Χ^2^ [2, N = 206) = 13.38, *p* = 0.004) [suicidal behaviors (Χ^2^ (3, N = 206) = 14.55, *p* = 0.002). Although these findings are of clinical interest, the limited sample size within subgroups may have reduced the statistical power of the analysis; therefore, the observed significance should be interpreted cautiously.

Group differences in psychiatric comorbidities are reported in Tables S1 and S2 (Supplementary Materials).

The results of the group comparisons regarding ACEs [adverse childhood experiences] are reported in Table 4 and Table 5.

These findings may indicate a potential association between BIF/ID and experiences of neglect; however, given the small number of participants within subgroups, these results should be considered preliminary, as limited subgroup sizes may have constrained the robustness of the statistical estimates.

## 4. Discussion

The clinical sample in this study has a mean age of 14.9 years, with approximately two-thirds (70.4%) of the patients being female. This distribution is consistent with epidemiological data from the pandemic and post-pandemic periods, which reported an increase in psychiatric emergency department admissions, particularly among patients aged 12 to 17 years [CDC, 2020] and among females [30,31].

In our ASD-HF subgroup, a male predominance was not observed (ASD-HF: males = 31.8%, females = 68.2%), which contrasts with the male predominance typically reported in epidemiological studies of the broader ASD population (males = 55.6%). It is well-established that the average male-to-female ratio in ASD is approximately 4:1 (4.34:1 in Europe and 4.28:1 in North America). This ratio increases to approximately 7–10:1 in cases of high-functioning ASD or Asperger’s syndrome and decreases to around 2:1 among individuals with moderate to severe intellectual disability [32,33]. Moreover, it is known that the male-to-female ratio tends to increase as the severity of intellectual disability decreases [34]. The predominance of female patients in our cohort of adolescents with high-functioning autism spectrum disorder may be related to the greater use of camouflage and masking strategies in females. While these mechanisms can obscure autistic features during periods of clinical stability, their collapse during acute psychopathological decompensation may lead to a more overt expression of core autism characteristics, facilitating diagnostic recognition and potentially explaining the observed female overrepresentation in psychiatric emergency settings.

In terms of overall functioning, the ASD patient group reported the lowest scores on the C-GAS scale 33.75 (±10.45), compared to the other groups, with a statistically significant difference relative to the N-ASD-N-BIF/ID group (mean score of 37.98 ± 8.17).

Within the ASD group, this finding is particularly pronounced in the ASD-BIF/ID subgroup 31.16 (±10.991), compared to patients in the N-ASD/N-BIF/ID group.

Several clinical studies have identified positive associations between cognitive abilities and adaptive functioning in autistic children and adolescents [35,36]. However, this result may also reflect the more frequent use of social camouflage among ASD patients without cognitive impairment. Social camouflage is frequently adopted as a coping strategy in response to social isolation, perceived social skill deficits, and low self-esteem, potentially masking functional difficulties [37]. Although it may initially promote apparent adaptation, it has been linked to poorer long-term mental health outcomes and reduced functioning [38,39]. Camouflaging behaviors have been associated with increased internalizing symptoms, identity difficulties [40], and a heightened risk of suicidal behaviors [41,42]. Indeed, camouflaging autistic traits is considered a transdiagnostic risk factor for lifetime suicidality within the framework of the Integrated Motivational-Volitional Model of Suicide [43,44].

In interpreting these findings, particular attention was paid to their clinical relevance, given the limited statistical power of some subgroup comparisons.

Our analyses highlighted differences in suicidal ideation and behaviours across clinical subgroups (Table 2 and Table 3) suggesting that, in terms of suicidal ideation and behaviors, ASD-HF patients more closely resemble the N-ASD–N-BIF/ID group than the ASD-BIF/ID or BIF/ID groups. This pattern may reflect the combination of heightened self-awareness, emotional dysregulation, and co-occurring psychiatric symptoms in high-functioning individuals on the spectrum, which could underlie an increased risk of suicidality, consistent with previous literature [18,21,45,46]. The lower prevalence of suicidal ideation observed in the BIF/ID group should nevertheless be interpreted cautiously. Rather than indicating a genuinely reduced risk, this finding may reflect methodological challenges in detecting suicidality in individuals with borderline intellectual functioning or intellectual disability.

In the present study, suicidal ideation was assessed using the Columbia Suicide Severity Rating Scale (C-SSRS), administered with developmentally and linguistically adapted wording for adolescents with cognitive impairment, and supplemented by caregiver reports. Although this multi-informant approach was intended to enhance detection, key limitations remain. Adolescents with BIF/ID may still have difficulty conceptualizing, interpreting, and communicating internal experiences of suicidal thoughts, even when questions are simplified. In addition, caregivers may not always recognize or accurately interpret signs of emotional distress or emerging suicidal ideation in their children, particularly in the absence of overt behavioral changes. Therefore, this result should be interpreted with caution. [21,47,48,49].

Within the context of adverse childhood experiences (ACEs, Table 4 and Table 5), a notable presence of neglect—both physical and emotional—is observed in the BIF/ID patient group (25%) and, to a lesser extent, in the ASD-BIF/ID subgroup (15.8%).

Neglect is recognized as the most frequently reported form of child maltreatment [50]. Several studies have linked this behavior to elevated levels of chronic parental stress [51,52], which may stem from the emotional and physical burden associated with the need to provide continuous care for a child with a severe physical or mental illness [53,54].

In particular, parents of children with intellectual disabilities (ID) have been reported to experience higher stress levels compared to parents of typically developing children [55,56]. Furthermore, the presence of behavioral disorders in children is associated with an additional increase in parental stress and a heightened risk of maltreatment [57,58,59]. That said, in families of children with disabilities, the perception of adequate healthcare and social support is inversely correlated with parental stress and appears to exert a protective effect. Therefore, providing appropriate medical and social support, alongside psychoeducational interventions for family members, may be beneficial in these cases.

Compared to individuals without disabilities, young people with (ID) are more frequently exposed to abuse, neglect, social disadvantage, challenging family environments, stigma, and peer exclusion [23], and they exhibit higher rates of depressive symptoms [12,24]. These findings highlight the importance of assessing quality of life and the potential presence of depressive symptoms in adolescents with intellectual disabilities using appropriately tailored instruments. Given that such symptoms may be masked by limited verbal expression and the broader clinical profile, careful evaluation is essential to identify unmet needs and to guide the development of individualized therapeutic interventions.

A notable finding in our sample is the prevalence of comorbid neurodevelopmental disorder (Tables S1 and S2) including 33% with ADHD (nearly one-third). The high prevalence of neurodevelopmental disorders among patients admitted to psychiatric service suggests that atypical developmental trajectories may contribute to the onset of acute psychiatric conditions [60].

Analysis of comorbidity distribution revealed that ADHD was present in over half of the patients with BIF/ID (N = 20; 62%), a significantly higher rate compared to the N-ASD-N-BIF/ID group (N = 32; 24%), and occurred in 52,6% of ASD-BIF/ID patients (N = 10). The high overlap between ADHD, ASD, and BIF/ID supports the conceptualization of neurodevelopmental disorders as a unified construct, with individual diagnoses representing points on a broader spectrum marked by shared and overlapping features. Over the past decade, mounting evidence has indicated that childhood neurodevelopmental disorders—including intellectual disability, autism spectrum disorder, and ADHD—share specific genetic risk alleles, environmental risk factors, and potentially interacting or synergistic pathogenic mechanisms [61].

The vast majority of the patients (82%) in our sample have an associated diagnosis within the bipolar spectrum disorder. The particularly high prevalence of bipolar spectrum disorders observed in the sample represents a considerable limitation to the generalizability of the study and may reflect a referral bias, as our center serves as a regional referral unit for complex psychiatric disorders and psychopharmacology, with a specific expertise in the diagnosis and management of bipolar disorders, including the initiation of lithium therapy, which in Italy is authorized exclusively for patients with a confirmed diagnosis of bipolar disorder. On the other hand, epidemiological data on psychiatric comorbidities in units exclusively dedicated to adolescent psychiatric emergencies are still rather scarce and heterogeneous. Nevertheless, our findings may be consistent with recent epidemiological data indicating an increase in pediatric emergency department visits for conditions such as substance use, manic episodes, and acute psychotic episodes, which may be related to underlying bipolarity [62]. Bipolar disorders are consistently represented across all groups; however, it is noteworthy that 86.4% of patients with high-functioning ASD (ASD-HF)present with comorbid bipolar disorder. This finding supports previous studies reporting that individuals with high-functioning ASD are at increased risk to developing mood disorders [63,64,65,66].

The observed correlation between early-onset bipolar disorder and autism spectrum disorder may not be incidental. Rather, it could represent a phenotypic outcome along specific developmental trajectories shaped by at least partially overlapping genetic patterns [67,68]. Indeed, some authors suggest that early-onset bipolar disorder itself could be conceptualized as a neurodevelopmental disorder, particularly due to developmental dimensions such as emotional dysregulation and cyclothymic temperament expression [69,70,71,72]. Recent studies indicate that the prevalence of bipolar disorder in pediatric ASD populations ranges from 2% to 10%, with higher rates in ASD patients without cognitive impairment and increasing from age 5 to 18. In adulthood, prevalence appears higher, reaching up to 40% in individuals with ASD and up to 66% in those with Asperger’s syndrome [73,74,75,76,77]. In any case, the comorbidity of ASD and bipolar disorder represents an important area for developmental research. Further studies are needed to clarify the precise prevalence of this condition, and longitudinal investigations are especially valuable to understand the developmental trajectory of this subgroup of patients.

The higher prevalence of dysfunctional personality traits in the Non-ASD/Non-BIF/ID group and the ASD-HF subgroup suggests differing trajectories of personality development and emotional regulation across neurodevelopmental profiles. Adolescents without neurodevelopmental disorders may show greater psychological insight, facilitating the recognition of maladaptive features such as affective instability, impulsivity, and interpersonal difficulties [78,79]. Similarly, high-functioning ASD individuals may display rigid cognitive and emotional patterns and difficulties in mentalizing, producing behaviors that resemble personality pathology, particularly within borderline and obsessive–compulsive spectra [80]. By contrast, the lower prevalence observed in BIF/ID and ASD-BIF/ID adolescents may reflect limited introspection, reduced emotional differentiation, or diagnostic overshadowing, compounded by assessment tools with reduced sensitivity in cognitively impaired populations [81,82]. These findings underscore the complex interplay between neurocognitive functioning, emotional regulation, and personality, highlighting the need for developmentally sensitive, neurodiversity-informed approaches to personality assessment in adolescent psychiatric settings. Take together, these findings should be interpreted within the methodological constraints of an exploratory retrospective study conducted in a tertiary referral setting.

## 5. Limits of the Study

This study has several limitations. Its cross-sectional, single-center and retrospective design restricts causal inference and prevents analysis of developmental trajectories over time. The relatively small sample size, especially within diagnostic subgroups, may also limit statistical power and generalizability. Moreover, although standardized assessment tools were used consistently across participants, they were not specifically validated for autistic or intellectually disabled populations, potentially reducing their sensitivity to the distinctive psychopathological profiles of these groups. This limitation may have influenced the sensitivity of group comparisons, particularly in detecting internalizing symptoms and suicidal ideation in adolescents with cognitive impairment. Another notable limitation of the study is the particularly high prevalence of bipolar spectrum disorders within the sample, which may reduce the generalizability of the findings. As highlighted in the discussion, this high prevalence may partly reflect a referral bias, given that our center serves as a specialized regional facility for complex psychiatric conditions and psychopharmacological treatment. The team has particular expertise in diagnosing and managing bipolar disorders, including the administration of lithium therapy, which in Italy is legally restricted to patients with a confirmed bipolar diagnosis.

A relevant limitation of the present study is also the unequal distribution of participants across diagnostic groups, with the N-ASD–N-BIF/ID group being substantially larger than the other groups. However, this imbalance reflects the natural case mix of patients admitted to our adolescent psychiatric emergency unit during the study period, considering that we included a consecutive, unselected sample of adolescents aged 11–18 years admitted to our unit over a time period of 2 years. Indeed, the distribution observed is consistent with the routine clinical flow of referrals to emergency psychiatric services, where neurotypical adolescents without autism spectrum disorder or borderline intellectual functioning/intellectual disability constitute the majority of admissions. While this uneven group size may limit the comparability and statistical power of between-group analyses, it also enhances the ecological validity of the findings by accurately representing real-world clinical practice.

Despite their clinical relevance, our results should be interpreted with caution due to the limited sample sizes of the subgroups, which may have reduced statistical power and increased the risk of type II errors. In particular, comparisons between the ASD-BIF/ID and BIF/ID groups were the most affected by limited statistical power. Nonetheless, the differential patterns observed across ASD-HF, ASD-BIF/ID, BIF/ID, and N-ASD–N-BIF/ID subgroups underscore the importance of careful suicide risk assessment tailored to cognitive and diagnostic profiles. Future research with larger, multicenter cohorts and longitudinal designs is warranted to better characterize the developmental trajectories and underlying mechanisms of suicidality in adolescents with neurodevelopmental disorders.

## 6. Conclusions

This study highlights the complex clinical profile of adolescents admitted to psychiatric emergency services, with a high prevalence of neurodevelopmental disorders, including ASD and BIF/ID, and frequent comorbidity with bipolar spectrum disorders. Our findings suggest that suicidal ideation and behaviors are not uniformly distributed across diagnostic subgroups: adolescents with high-functioning ASD exhibit patterns more similar to neurotypical peers than to individuals with cognitive impairments, while suicidality in adolescents with BIF/ID may be underestimated due to methodological and cognitive constraints.

The study also indicates the interplay between cognitive functioning, emotional regulation, social camouflage, and maladaptive personality traits, as well as the impact of adverse childhood experiences and family stress on clinical outcomes. These factors point to the need for comprehensive, developmentally sensitive, and neurodiversity-informed approaches to assessment and intervention.

Despite methodological limitations—including the retrospective and cross-sectional design, limited subgroup sizes, high prevalence of bipolar disorders, and the use of standardized tools not specifically validated for autistic or intellectually impaired populations—the present findings may contribute to understanding risk profiles and functional impairments in this population.

Overall, our results suggest the importance of early identification and careful monitoring of psychiatric symptoms, including suicidality, in adolescents with neurodevelopmental disorders. Tailored interventions that integrate medical, social, and psychoeducational support for patients and their families are recommended. Future longitudinal and multicenter studies are needed to confirm these hypothesis-generating findings and to clarify developmental trajectories and mechanisms underlying acute psychiatric crises in this populations.

## Figures and Tables

**Table 1:** Descriptive analysis of the cohort.

VARIABLE	MEAN ± sd
Age [years]	14.91 ± 1.771
Admission CGI-S	6.8 ± 0.475
Hospital discharge CGI-S	5.51 ± 0.791
C-GAS	36.92 ± 8.91
Average length of hospitalization [days]	14.78 ± 9.743
VARIABLE	FREQUENCY [%/%]
Female/Male	145/61 [70.4/29.6%]

CGI-S: Clinical Global ImpressionSeverity; C-GAS: Children’s Global Assessment Scale.

**Table 2:** Group comparison in suicidality and non-suicidal self injuries (NNSI).

	ASD (N = 36)	BIF/ID (N = 39)	N-ASD-N-BIF/ID (N = 132)	Pearson Chi Square (Value; df); *p*	Post Hoc Z Test
Self-injurious Behaviors No/Yes (%)	22/19 (53.7/46.3)	14/18 (43.8/56.39	59/74 (44.4/55.6)	(1.178;2); ns	-
Suicidal Ideation No/Yes (%)	28/13 (68.3/31.7)	25/7 (78.1/21.9)	68/65 (51.1/48.9)	(9.685;2); 0.008	BIF/ID ≠ N-ASD-N-BIF/ID
Suicidal Behaviours No/Yes (%)	33/8 (80.5/19.5)	27/5 (78.1/21.9)	79/54 (59.4/40.69)	(11.281;2); 0.004	ASD ≠ N-ASD-N-BIF/ID BIF/ID ≠ N-ASD-N-BIF/ID

ASD: autism spectrum disorder; ASD BIF/ID: autism spectrum disorder with borderline intellectual functioning or intellectual disability; N-ASD-N-BIF/ID: non autism spectrum disorder, Non Borderline intellectual functioning, Non intellectual disabilities; ns: not significant.

**Table 3:** Group comparison in suicidality and non-suicidal self injuries (NNSI) (ASD subtypes).

	ASD-HF (N = 21)	ASD-BIF/ID (N = 15)	BIF/ID (N = 39)	N-ASD-N-BIF/ID (N = 132)	Pearson Chi Square (Value; df); *p*	Post Hoc Z Test
Self-injurious Behaviours No/Yes (%)	9/13 (40.9/59.1)	13/6 (68.4/31.6)	14/18 (43.8/56.3)	59/74 (44.4/55.6)	(4.281;3); ns	-
Suicidal Ideation No/Yes (%)	12/10 (54.5/45.5)	16/3 (84.2/15.8)	25/7 (78.1/21.9)	68/65 (51.1/48.9)	(13.387;3); 0.004	ASD-BIF/ID e BIF/ID ≠ N-ASD-N-BIF/ID
Suicidal Behaviours No/Yes (%)	15/7 (68.2/31.8)	18/1 (94.7/5.3)	27/5 (78.1/21.9)	79/54 (59.4/40.6)	(14.557;3); 0.002	ASD-HF e ASD-BIF/ID ≠ N-ASD-N-BIF/ID

ASD: autism spectrum disorder; HF-ASD: high functioning ASD; ASD BIF/ID: autism spectrum disorder with borderline intellectual functioning or intellectual disability; N-ASD-N-BIF/ID: non autism spectrum disorder, non Borderline intellectual functioning, non intellectual disabilities; ns: not significant.

**Table 4:** Group comparison in qualitative features: direct and indirect adverse childhood experiences [ACE].

	ASD (N = 41)	BIF/ID (N = 32)	N-ASD-N-BIF/ID (N = 133)	Pearson Chi Square (Value; df; *p*)	Post Hoc Z Test
Sexual abuse No/Yes (%)	41/0 (100/0)	32/0 (100/0)	130/3 (97.7/2.3)	(1.67;2); ns	-
Recurring physical maltreatment No/Yes (%)	40/1 (97.6/2.4)	29/3 (90.6/9.4)	129/4 (97/3)	(3.08;2); ns	-
Recurring psychological maltreatment No/Yes (%)	40/1 (97.6/2.4)	29/3 (90.6/9.4)	127/6 (95.5/4.5)	(1.96;2); ns	-
Physical and emotional neglect No/Yes (%)	38/3 (92.7/7.3)	24/8 (75/25)	119/14 (89.5/10.5)	(6.18;2); 0.045	BIF/ID ≠ ASD and N-ASD-N-BIF/ID
Witnessed violence No/Yes (%)	39/2 (95.1/4.9)	32/0 (100/0)	126/7 (94.7/5.3)	(1.74;2); ns	-
Alcohol and substance abuse No/Yes (%)	39/2 (95.1/4.9)	29/3 (90.6/9.4)	121/12 (91/9)	(0.77;2); ns	-
Psychiatric illness No/Yes (%)	38/3 (92.7/7.3)	29/3 (90.6/9.4)	109/24 (82/18)	(3.7;2); ns	-
Single parent or orphan No/Yes (%)	38/3 (92.7/7.3)	28/4 (87.5/12.5)	124/9 (93.2/6.8)	(1.18;2); ns	-
Jailed parent No/Yes (%)	41/0 (100/0)	30/2 (93.8/6.3)	127/6 (95.5/4.5)	(2.27;2); ns	-

ASD: autism spectrum disorder; ASD BIF/ID: autism spectrum disorder with borderline intellectual functioning or intellectual disability; N-ASD-N-BIF/ID: non autism spectrum disorder, non borderline intellectual functioning, non intellectual disabilities; ns: not significant.

**Table 5:** Group comparison in qualitative features: direct and indirect adverse childhood experiences (ACE) (ASD subtypes).

	ASD-HF (N = 22)	ASD-BIF/ID (N = 19)	BIF/ID (N = 32)	N-ASD-N-BIF/ID (N = 133)	Pearson Chi Square (Value; df); *p*	Post Hoc Z Test
Sexual abuse No/Yes (%)	22/0 (100/0)	19/0 (100/0)	32/0 (100/0)	130/3 (97.7/2.3)	(1.67;3); ns	-
Recurring physical maltreatment No/Yes (%)	21/1 (95.5/4.5)	19/0 (100/0)	29/3 (90.6/9.4)	129/4 (97/3)	(3.6;3); ns	-
Recurring psychological maltreatment No/Yes (%)	21/1 (95.5/4.5)	19/0 (100/0)	29/3 (90.6/9.4)	127/6 (95.5/4.5)	(2.42;3); ns	-
Physical and emotional neglect No/Yes (%)	22/0 (100/0)	16/3 (84.2/15.8)	24/8 (75/25)	119/14 (89.5/10.5)	(8.2;3); 0.036	BIF/ID ≠ ASD-HF and N-ASD-N-BIF/ID
Witnessed violence No/Yes (%)	20/2 (90.9/9.1)	19/0 (100/0)	32/0 (100/0)	126/7 (94.7/5.3)	(3.75;3); ns	-
Alcohol and substance abuse No/Yes (%)	22/0 (100/0)	17/2 (89.5/10.5)	29/3 (90.6/9.4)	121/12 (91/9)	(2.26;3); ns	-
Psychiatric illness No/Yes (%)	20/2 (90.9/9.1)	18/1 (94.7/5.3)	29/3 (90.6/9.4)	109/24 (82/18)	(3.83;3); ns	-
Single parent or orphan No/Yes (%)	20/2 (90.9/9.1)	18/1 (94.7/5.3)	28/4 (87.5/12.5)	124/9 (93.2/6.8)	(1.406;3); ns	-
Jailed parent No/Yes (%)	22/0 (100/0)	19/0 (100/0)	30/2 (93.8/6.3)	127/6 (95.5/4.5)	(2.27;3); ns	-

ASD: autism spectrum disorder; HF-ASD: high functioning ASD; ASD BIF/ID: autism spectrum disorder with borderline intellectual functioning or intellectual disability; N-ASD-N-BIF/ID: non autism spectrum disorder, non Borderline intellectual functioning, non intellectual disabilities; ns: not significant.

## Data Availability

Data available in a publicly accessible repository; the original data presented in the study are openly available in the Zenodo repository at: https://doi.org/10.5281/zenodo.18172417 accessed on 7 January 2026.

## Supplementary Materials

The following supporting information can be downloaded at: https://www.mdpi.com/article/10.3390/brainsci16030250/s1. Table S1: Group comparison in Comorbidities; Table S2: Group comparison in Comorbidities (ASD subgroups).
