# Supplementary material for: Autism, Intellectual Disability and Suicide Risk in Adolescent Psychiatric Emergencies: A Two-Year Retrospective Cohort Study
Source: Brain Sci. 2026 Feb 24;16(3):250. doi: 10.3390/brainsci16030250 (PMC13024299; doi:10.3390/brainsci16030250)
Supplement: Supplementary file 1 [file brainsci-16-00250-s001.zip › brainsci-4098371-supplementary.pdf]

Table S1. Group comparison in Comorbidities.

	ASD (N=41)	BIF/ID (N=32)	N-ASD-N-BIF/ID (N=133)	Pearson Chi square (valore; df); p	Post hoc Z test
ADHD No/Yes (%)	25/16 (61/39)	12/20 (37/62)	101/32 (76/24)	(18.07;2); 0.000	BIF/ID ≠ N-ASD-N-BIF/ID
Bipolar disorder No/Yes (%)	7/34 (17.1/82.9)	5/27 (15.6/84.4)	24/109 (18/82)	(0.111;2); ns	-
Psychotic disorders No/Yes (%)	27/14 (65.9/34.1)	24/8 (75/25)	126/7 94,7/5,3)	(25,351;2); 0.000	ASD e BIF/ID ≠ N-ASD-N-BIF/ID
Anxiety No/Yes (%)	14/27 (34.1/65.9)	11/21 (34.4/65.6)	13/120 (9.8/90.2)	(18.763;2); 0.000	ASD e BIF/ID≠ N-ASD-N-BIF/ID
Disruptive disorders No/Yes (%)	30/11 (73.2/26.8)	14/18 (43.8/56.3)	104/29 (78.2/21.8)	(15.174;2); 0.001	ASD e N-ASD-N-BIF/ID ≠ BIF/ID
Learning disorders No/Yes (%)	32/9 (78/22)	24/8 (75/25)	111/22 (83,5/16,5)	(1.5;2); ns	-
Depression No/Yes (%)	25/16 (61/39)	15/17 (46,9/53.1)	53/80 (39.8/60.2)	(5.694;2); ns	-
Obsessive compulsive disorder No/Yes (%)	33/8 (80.5/19.5)	28/4 (87.5/12.5)	112/21 (84,2/15.8)	(0.672;2); ns	-
Tic disorder No/Yes (%)	39/2 (95.1/4.9)	27/5 (84.4/15.6)	129/4 (97/3)	(8.146;3); ns	-
Traumatic disorder No/Yes (%)	26/15 (63.4/36.6)	14/18 (43.8/56.3)	69/64 (51.9/48.1)	(2,95;2); ns	-
Personality disorders No/Yes (%)	24/17 (58.5/41.5)	21/11 (65.6/34.4)	40/92 (30.3/69.7)	(19.395;2); 0.000	ASD e BIF/ID ≠ N-ASD-N-BIF/ID
Dissociative disorder No/Yes (%)	38/3 (92.7/7.3)	31/1 (96.9/3.1)	127/6 (95.5/4.5)	(0.779;2); ns	-
Somatic complaints No/Yes (%)	35/6 (85.4/14.6)	25/7 (78.1/21.9)	100/33 (75.2/24.8)	(1.876,0;2); ns	-
Nutrition disorders No/Yes (%)	33/8 (80.5/19.5)	22/10 (68.8/31.3)	84/49 (63.2/36.8	(4.31;2); ns	-
Addiction No/Yes (%)	37/4 (90.2/9.8)	29/3 (90.6/9.4)	113/20 (85/15)	(1.231;2); ns	-
Sleep disorder No/Yes (%)	39/2 (95.1/4.9)	31/1 (96.9/3.1)	119/14 (89.5/10.5)	(2.636;2); ns	-
Evcacuation disorders No/Yes (%)	40/1 (97.6/2.4)	31/1 (96.9/3.1)	133/0 (100/0)	(3.788;2); ns	-

ASD: autism spectrum disorder; ASD BIF/ID: autism spectrum disorder with borderline intellectual functioning or intellectual disability; N-ASD-N-BIF/ID :non autism spectrum disorder, non borderline intellectual functioning, non intellectual disabilities; ns not significant.

Table S2. Group comparison in Comorbidities (ASD subgroups)

	ASD-HF (N=25)	ASD-BIF/ID (N=12)	BIF/ID (N=32)	N-ASD-N-BIF/ID (N=133)	Pearson Chi square (valore; df); p	Post hoc Z test
ADHD No/Yes (%)	16/6 (72.7/27.3)	9/10 (47.4/52.6)	12/20 (37/62)	101/32 (76/24)	(21,03;3); 0.000	BIF/ID ≠ N-ASD-N-BIF/ID
Bipolar disorder No/Yes (%)	3/19 (13.6/86.4)	4/15 (21.1/78.9)	5/27 (15.6/84.4)	24/109 (18/82)	(0.499;3); ns	-
Psychotic disorders No/Yes (%)	18/4 (81.8/18.2)	9/10 (47.4/52.6)	24/8 (75/25)	126/7 94,7/5,3)	(35.354;3); 0.000	ASD e BIF/ID ≠ N-ASD-N-BIF/ID
Anxiety No/Yes (%)	6/16 (34.1/65.9)	8/11 (41.1/57.9)	11/21 (34.4/65.6)	13/120 (9.8/90.2)	(20.254;3); .000	ASD e BIF/ID≠ N-ASD-N-BIF/ID
Disruptive disorders No/Yes (%)	19/3 (86.4/13.6)	11/8 (57.9/42.1)	14/18 (43.8/56.3)	104/29 (78.2/21.8)	(19.259:3); 0.000	ASD e N-ASD-N-BIF/ID ≠ BIF/ID
Learning disorders No/Yes (%)	18/4 (81.8/18.2)	14(5 (73.7/26.3)	24/8 (75/25)	111/22 (83,5/16,5)	(1.046;3); ns	-
Depression No/Yes (%)	11/11 (50/50)	14/5 (73.7/26.3)	15/17 (46,9/53.1)	53/80 (39.8/60.2)	(8.003;3); 0.046	ASD BIF/ID ≠ N-ASD-N-BIF/ID
Obsessive compulsive disorder No/Yes (%)	17/5 (77.3/22.7)	16/3 (84.2/15.8)	28/4 (87.5/12.5)	112/21 (84,2/15.8)	(1.036;3); ns	-
Tic disorder No/Yes (%)	22/0 (100/0)	17/2 (89.5/10.5)	27/5 (84.4/15.6)	129/4 (97/3)	(10.38;3); 0.016	BIF/ID ≠ N-ASD-N-BIF/ID
Traumatic disorder No/Yes (%)	13/9 (59.1/40.9)	13/6 (68.4/31.6)	14/18 (43.8/56.3)	69/64 (51.9/48.1)	(3.306;3); ns	-
Personality disorders No/Yes (%)	7/15 (31.8/68.2)	17/2 (89.5/10.5)	21/11 (65.6/34.4)	40/92 (30.3/69.7)	(33.358;3); 0.000	ASD e BIF/ID ≠ N-ASD-N-BIF/ID
Dissociative disorder No/Yes (%)	21/1 (95.5/4.5)	17/2 (89.5/10.5)	31/1 (96.9/3.1)	127/6 (95.5/4.5)	(1.569;3); ns	-
Somatic complaints No/Yes (%)	19/3 (86.4/13.6)	16/3 (84.2/15.8)	25/7 (78.1/21.9)	100/33 (75.2/24.8)	(1.9;3); ns	-
Nutrition disorders No/Yes (%)	16/6 (72.7/27.3)	17/2 (89.5/10.5)	22/10 (68.8/31.3)	84/49 (63.2/36.8	(5.6:3); ns	-
Addiction No/Yes (%)	21/1 (95.5/4.5)	16/3 (84.2/15.8)	29/3 (90.6/9.4)	113/20 (85/15)	(2.363;3); ns	-
Sleep disorder No/Yes (%)	21/1 (95.5/4.5)	18/1 (94.7/5.3)	31/1 (96.9/3.1)	119/14 (89.5/10.5)	(2.643); ns	-

Evacuation disorders No/Yes (%)	22/0 (100/0)	18(1 (94.7/5.3)	31/1 (96.9/3.1)	133/0 (100/0)	(8.705;3); ns	-

ASD: autism spectrum disorder; HF-ASD: high functioning ASD; ASD BIF/ID: autism spectrum disorder with borderline intellectual functioning or intellectual disability; N-ASD-N-BIF/ID :non autism spectrum disorder, non borderline intellectual functioning, non intellectual disabilities ; ;ns not significant.
